# Suppression of nuclear spin bath fluctuations in self-assembled quantum dots induced by inhomogeneous strain

**DOI:** 10.1038/ncomms7348

**Published:** 2015-02-23

**Authors:** E.A. Chekhovich, M. Hopkinson, M.S. Skolnick, A.I. Tartakovskii

**Affiliations:** 1 Department of Physics and Astronomy, University of Sheffield, Sheffield S3 7RH, UK; 2 Department of Electronic and Electrical Engineering, University of Sheffield, Sheffield S1 3JD, UK

## Abstract

Interaction with nuclear spins leads to decoherence and information loss in solid-state electron-spin qubits. One particular, ineradicable source of electron decoherence arises from decoherence of the nuclear spin bath, driven by nuclear–nuclear dipolar interactions. Owing to its many-body nature nuclear decoherence is difficult to predict, especially for an important class of strained nanostructures where nuclear quadrupolar effects have a significant but largely unknown impact. Here, we report direct measurement of nuclear spin bath coherence in individual self-assembled InGaAs/GaAs quantum dots: spin-echo coherence times in the range 1.2–4.5 ms are found. Based on these values, we demonstrate that strain-induced quadrupolar interactions make nuclear spin fluctuations much slower compared with lattice-matched GaAs/AlGaAs structures. Our findings demonstrate that quadrupolar effects can potentially be used to engineer optically active III-V semiconductor spin-qubits with a nearly noise-free nuclear spin bath, previously achievable only in nuclear spin-0 semiconductors, where qubit network interconnection and scaling are challenging.

## Abstract (web summary)

Decoherence of the nuclear spin bath causes decoherence of electron spin qubits in the solid state. Here, Chekhovich *et al.* use spin-echo measurements to demonstrate the suppression of nuclear spin fluctuations in semiconductor quantum dots via strain-induced quadrupolar interactions.

## Graphical abstract

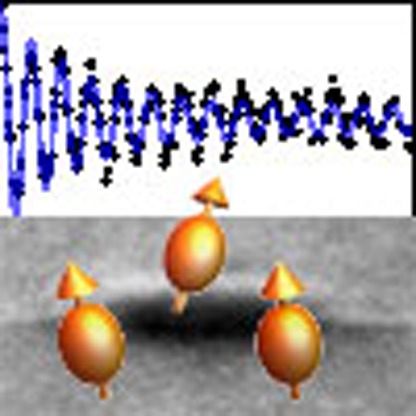

Quantum dots (QDs) in III-V semiconductors have many favourable properties for applications in quantum information processing^1^,^2^,^3^,^4^. Self-assembled dots are particularly promising because of their strong interaction with light offering excellent optical interfacing, manipulation at ultrafast speeds and advanced manufacturing technology^5^,^6^,^7^,^8^. However, all atoms of groups III and V have non-zero nuclear magnetic moments. Thus, instead of an ideal two-level quantum system, the spin of a single electron in a QD is described by the so-called ‘central spin’ problem^9^,^10^,^11^,^12^, where the electron (central) spin is subject to magnetic interaction with an ensemble of 10^4^–10^6^ nuclear spins. This hyperfine interaction results in decoherence, that is, decay of the phase information encoded in electron spin^1^,^2^,^3^,^4^,^5^,^6^,^7^,^8^,^9^,^10^,^11^,^12^.

Hyperfine-induced decoherence can be greatly reduced by applying static magnetic field and refocusing control pulses inducing electron spin echo. With this technique, very long electron qubit coherence times of ~200 μs were demonstrated in lattice-matched GaAs/AlGaAs QDs^1^. However, the effect of nuclei can not be eliminated completely because of the presence of nuclear–nuclear (dipole–dipole) magnetic interactions, which cause spin exchange flip-flops of nuclei, that is, nuclear spin bath decoherence. Such flip-flops induce quasi-random fluctuating magnetic fields acting on electron spin and causing its decoherence (‘spectral diffusion’ process^10^,^11^). It is thus evident that understanding the nuclear spin coherence is crucial for predicting the coherence properties of the central spin. Furthermore, it is predicted that large nuclear quadrupolar interactions (QIs) present in strained self-assembled dots^13^ can suppress the nuclear flip-flops resulting in extended electron spin coherence^14^. However, this possibility is little explored^15^, mainly due to the lack of reliable data on nuclear spin coherence in self-assembled dots.

Here we demonstrate pulsed nuclear magnetic resonance (NMR) of as few as 10^4^–10^5^ quadrupolar spins in individual inhomogeneously strained InGaAs/GaAs QDs. We probe nuclear coherence by measuring spin-echo decay times *T*_2_, which are found to be a factor of ~5 longer compared with lattice-matched (unstrained or homogeneously strained) GaAs/AlGaAs structures—direct evidence of nuclear spin flip-flop suppression induced by inhomogeneous QI. We then show that the nuclear flip-flop times *T*_2,ff_ (relevant for electron spin decoherence) are larger than spin-echo *T*_2_ times, but can be estimated using a first-principles model^16^,^17^. We conclude that the flip-flops of nuclei in spin states other than ±1/2 are completely frozen. For ±1/2 spin states, there is a difference between isotopes: while arsenic is frozen, the flip-flops of gallium and indium are possible but with *T*_2,ff_~5 ms, which is a factor of ~3–8 slower than in lattice-matched structures.

The unusual behaviour of arsenic is explained by additional inhomogeneous QI arising from random alloy mixing of gallium and indium atoms^18^. Such atomic-scale disorder opens a new prospect for using the excellent properties of III–V QDs to build nuclear-spin-noise free solid-state qubits: this can now be done without resorting to materials with zero nuclear spin (for example, isotopically pure ^28^Si and ^12^C)^19^,^20^,^21^, which have inferior optical properties, hampering on-chip integration of a large number of qubits.

## Results

### Nuclear quadrupolar effects in self-assembled QDs

Our experiments were performed on individual neutral QDs in InGaAs/GaAs samples, grown by strain-driven self-assembly using molecular beam epitaxy. The sample was placed in an optical helium-bath cryostat (*T*=4.2 K). Magnetic field *B*_z_ up to 8 T was applied parallel to the sample growth axis (*Oz*) and light propagation direction (Faraday geometry). The structures were investigated using optically detected NMR techniques, which extend the concepts reported in our recent work^13^,^22^. Radio-frequency (rf) fields *B*_rf_ perpendicular to *B*_z_ are induced by a minicoil wound around the sample (see further details in Methods, Supplementary Figs 1 and 2, and Supplementary Note 1).

**Figure 1:**
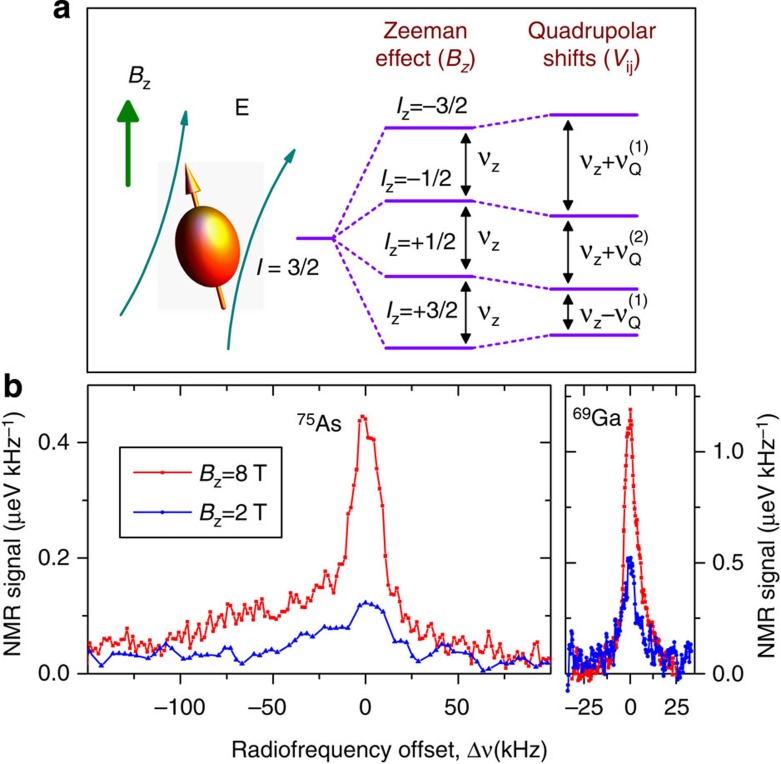
Nuclear quadrupolar effects in strained self-assembled quantum dots. (**a**) Schematic representation of a nuclear spin *I*=3/2 that has a non-spherical symmetry resulting in a non-zero nuclear quadrupolar moment. Magnetic field *B*_z_ lifts the fourfold degeneracy resulting in a Zeeman ladder of spin states. The dipole-allowed NMR transitions *I*_z_↔*I*_z_±1 occur at the same Larmor frequency *ν*_Z_. When electric field **E** has non-zero gradients 
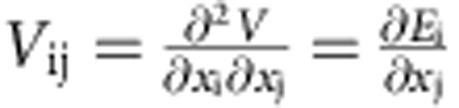
 (induced, for example, by strain) the NMR frequencies are modified by the first- and second-order shifts 
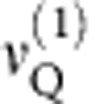
, 
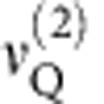
 with 
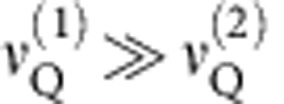
. (**b**) Central transition spectra of ^69^Ga and ^75^As measured in an InGaAs QD using continuous wave inverse techniques^13^ at *B*_z_=2 T (blue lines) and 8 T (red lines). The radiofrequency offset Δ*ν*=*ν*−*ν*_*Z*_ is calculated with respect to the Zeeman frequency *ν*_*Z*_=*B*_*z*_*γ*/(2*π*), where *γ*/(2*π*)≈7.33 MHz T^−1^ for ^75^As and 10.3 MHz T^−1^ for ^69^Ga. At lower magnetic field, the resonance peaks become weaker and broader, confirming that the linewidth is determined by the second-order shifts 
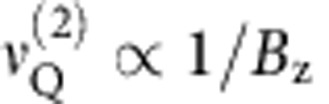
.

In this work, we study the four most abundant isotopes: ^69^Ga, ^71^Ga, ^75^As (spin *I*=3/2) and ^115^In (spin *I*=9/2), all possessing non-zero quadrupolar moments. The proportion of Ga/In in our dots is estimated as 0.76/0.24 (ref. 13). The energy level diagram of a quadrupolar nuclear spin is shown schematically in Fig. 1a for the case of *I*=3/2. Magnetic field *B*_z_ induces shifts proportional to ∝*I*_z_, so that all dipole-allowed NMR transitions (Δ*I*_z_=±1) appear at the same frequency *ν*_Z_. Electric field gradients (described by a second-rank tensor *V*_ij_, where *V* is the electrostatic potential) induce quadrupolar shifts proportional to 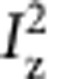 to first order of perturbation^23^. The resulting NMR frequency shifts 
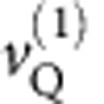
 are strongly inhomogeneous and are on the order of few MHz in InGaAs dots^13^,^24^. The central transition (CT) −1/2↔+1/2 is an exception, as it is affected by QI only to second order resulting in much smaller shifts 
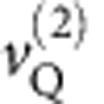
 on the order of tens to hundreds of kHz (ref. 23). The relatively small linewidths greatly simplify the experiments; thus in what follows we focus on spectroscopy of CTs only. In particular, selective pulsed NMR of CTs can be conveniently implemented by choosing the rf amplitude *B*_rf_ so that 
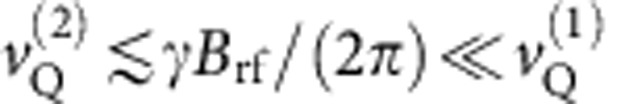
 (γ is the nuclear gyromagnetic ratio).

Figure 1b shows CT spectra of ^75^As and ^69^Ga measured using continuous-wave inverse NMR techniques^13^. At high-field *B*_z_=8 T, the ^69^Ga resonance consists of a narrow line (full-width at half-maximum=~9 kHz). The arsenic resonance is broader (full-width at half-maximum ~30 kHz) with additional asymmetric sidebands approximately 100 kHz broad. When magnetic field is reduced down to 2 T both resonances broaden and diminish in amplitude, as expected for a lineshape determined by second-order quadrupolar shifts 
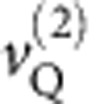
 (ref. 23). The significantly larger broadening of the ^75^As CT resonance is attributed to random intermixing of the group-III Ga and In atoms creating additional low-symmetry electric field gradients at arsenic sites^13^,^18^. As we demonstrate below, such random quadrupolar shifts result in pronounced suppression of dipolar nuclear flip-flops and extension of nuclear spin coherence times.

### Pulsed NMR spectroscopy of quadrupolar nuclei

**Figure 2:**
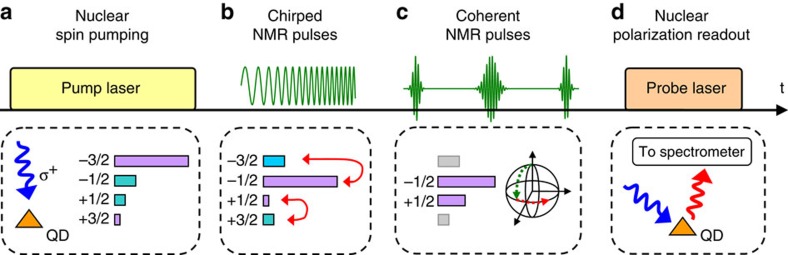
Optically detected pulsed NMR on central transitions (CTs) of quadrupolar nuclear spins in InGaAs quantum dots. Experimental cycle timing consists of four stages: (**a**) Optical pumping of a large (>50%) nuclear spin polarization^22^,^32^ is achieved by exciting the QD with a σ^+^ circularly polarized light. As a result, most nuclei are initialized into the *I*_*z*_=−3/2 state (spin-state populations are shown with horizontal bars). (**b**) To enhance the NMR signal of the CT, the population of the *I*_z_=−1/2(+1/2) state is maximized (minimized). This is achieved by swapping the populations of the −3/2 and −1/2 states and +1/2 and +3/2 states using chirped radiofrequency pulses^36^ (shown with arrows). (**c**) An arbitrary sequence of radiofrequency pulses (for example, spin-echo sequence) is applied selectively exciting the CT subspace (*I*_z_=−1/2, +1/2 states). All pulse sequences are designed to align the final magnetization along the *Oz* axis making it optically detectable. (**d**) Quantum dot is excited with an optical probe pulse inducing photoluminescence, which is analysed with a double spectrometer in order to measure the polarization of the final nuclear spin state^22^,^32^.

**Figure 3:**
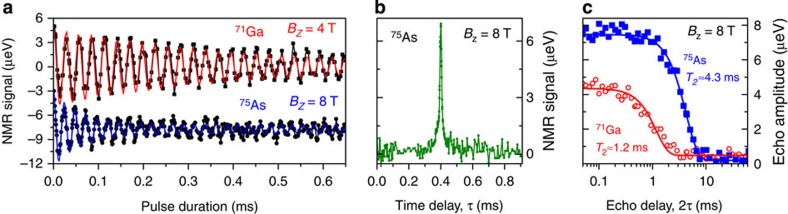
Coherent dynamics of the central transitions of quadrupolar nuclear spins in InGaAs quantum dots. (**a**) Rabi oscillations measurements. Nuclear spin polarization is measured as a function of the single rf pulse duration for ^71^Ga (top trace) and ^75^As (bottom trace). The ^75^As oscillations decay faster due to the stronger inhomogneous spectral broadening. (**b**) Hahn-echo measurement (90°−*τ*_0_−180°−*τ*−90° sequence with *τ*_0_=0.4 ms): a pronounced spin-echo signal is observed at *τ*=0.4 ms when the dephasing induced by inhomogeneous broadening is refocused. (**c**) Echo decay measurements at *B*_z_=8 T on ^71^Ga (circles) and ^75^As (squares): Spin-echo amplitude is plotted as a function of the total delay 2*τ* of the 90°−*τ*−180°−*τ*−90° sequence. Lines show Gaussian decay fitting 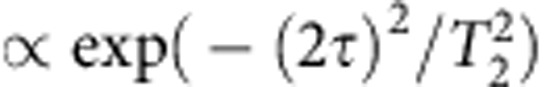 with decay times *T*_2_ characterizing the nuclear spin coherence.

The quadrupolar broadening of NMR spectra in Fig. 1b is inhomogeneous in character. It obscures the much weaker homogeneous broadening induced by the nuclear–nuclear interactions, which determine the nuclear spin coherence. In order to access the nuclear spin coherence, we use time-domain (pulsed) NMR^23^: the timing diagram of the pulsed NMR experiment is shown in Fig. 2 (further details on techniques can be found in Methods, Supplementary Figs 1 and 2, and Supplementary Note 1). We start with a Rabi nutation experiment where a single rf pulse of a variable duration *τ* is applied^25^. Rabi oscillations of nuclear polarization are clearly seen in Fig. 3a for ^71^Ga and ^75^As enabling the calibration of 90° and 180° rotation pulses. The decay of Rabi oscillations is due to dephasing caused by inhomogeneous spectral broadening (Fig. 1b). Such dephasing can be reversed using the Hahn echo sequence 90°−*τ*_0_−180°−*τ*−90°. The result of a measurement with a fixed delay *τ*_0_=0.4 ms and a variable *τ* are shown for ^75^As in Fig. 3b where as expected a pronounced spin echo is observed at *τ*=*τ*_0_.

**Figure 4:**
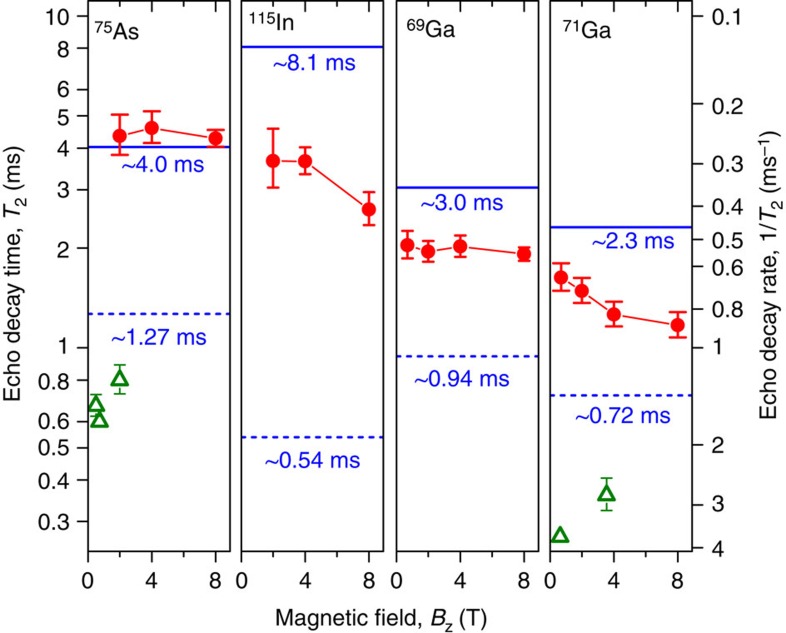
Nuclear spin-echo decay of central transitions. Echo decay times *T*_2_ (left scale) and corresponding decay rates 1/*T*_2_ (right scale) for four different isotopes as a function of *B*_z_. Circles—experiment on inhomogeneously strained InGaAs QDs (bars show 90% confidence intervals), triangles—measurements on lattice-matched GaAs/AlGaAs quantum wells and QDs (data taken from refs 25, 26, 27, 28, 29). Calculated *T*_2_ values^16^ are shown for the case of negligible second-order quadrupolar shifts 
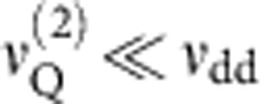
 as in lattice-matched GaAs/AlGaAs structures (dashed lines), and for the case of large inhomogeneous second-order quadrupolar shifts 
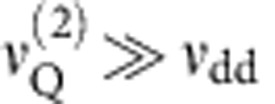
 resulting in complete suppression of nuclear flip-flops (solid lines).

We then turn to the spin-echo decay measurements (90°−*τ*−180°−*τ*−90° pulse sequence) where the evolution times *τ* before and after the 180° refocusing pulse are varied simultaneously. Figure 3c shows experimentally measured nuclear spin-echo amplitudes (symbols) as a function of the total delay time 2*τ* for ^71^Ga and ^75^As isotopes at *B*_z_=8 T. Experimental curves are well fitted by a Gaussian decay function (solid lines) with characteristic 1/*e* decay time *T*_2_≈1.18 ms for ^71^Ga and *T*_2_≈4.27 ms for ^75^As. The spin-echo sequence removes the effect of inhomogeneous spectral broadening, with the echo decay caused solely by nuclear–nuclear dipolar interactions^23^: *T*_2_ thus characterizes the coherence of the nuclear spin bath. We have repeated spin-echo measurements for all four studied isotopes at different magnetic fields *B*_z_. The resulting coherence times *T*_2_ (and corresponding decay rates 1/*T*_2_) are plotted in Fig. 4 by the circles. In addition, we have verified the reproducibility of our results by measuring *T*_2_ of ^75^As for another six individual dots from the same sample (see Supplementary Fig. 3 and Supplementary Note 2).

### Quadrupolar suppression of nuclear spin bath fluctuations

To examine the effect of inhomogeneous QI on the nuclear spin bath coherence, we first compare our experimental *T*_2_ times with previous nuclear spin echo measurements on lattice-matched GaAs/AlGaAs quantum wells (QWs) and dots. The data available for ^75^As (selective echo on CT in QWs^26^,^27^,^28^) and ^71^Ga (non-selective echo on QWs^29^ and QDs^25^) are shown in Fig. 4 by the triangles. It can be seen that the echo decay times in inhomogeneously strained self-assembled QDs are a factor of ~5–7 larger compared with unstrained or homogeneously strained lattice-matched structures. Such increase in *T*_2_ is due to suppression of nuclear spin flip-flops and provides direct evidence for the slow down of nuclear spin bath fluctuations in the presence of spatially inhomogeneous QI.

To quantify the effect of QI on the nuclear spin bath dynamics, we turn to more detailed analysis of our experimental results. At sufficiently large magnetic field *B*_z_>>10 mT, the interaction between any two nuclear spins *I* and *J* is described by the truncated dipole–dipole Hamiltonian^23^:


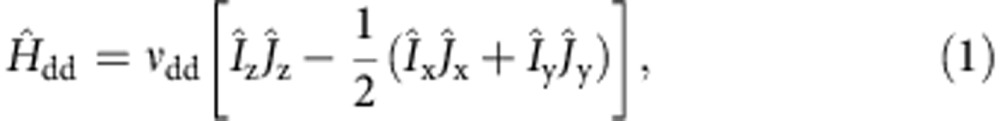


Where *Î* and *Ĵ* are spin operators, and the coupling strength *ν*_dd_ depends on nuclei type and mutual position (*ν*_dd_≲200 Hz in frequency units for nearest neighbours in InGaAs, and scales as ∝*r*^−3^ with internuclear distance *r*). The (*Î*_x_*Ĵ*_x_+*Î*_y_*Ĵ*_y_) term enables spin exchange flip-flops between nuclei *I* and *J*: (*I*_z_, *J*_z_) ↔ (*I*_z_±1, *J*_z_∓1), the process ultimately responsible for electron spin decoherence via spectral diffusion. A flip-flop can only happen if *I* and *J* are no more than a few unit cells apart (due to ∝*r*^−3^ scaling of *Ĥ*_dd_) and have similar Zeeman energies requiring them to be of the same isotope. If, however, these two nuclei are subject to significantly different quadrupolar shifts *ν*_Q,I_ and *ν*_Q,J_, so that |*ν*_Q,I_−*ν*_Q,J_|>>*ν*_dd_ the flip-flops will become energetically forbidden, resulting in a slow down of nuclear spin bath dynamics and potential increase in electron qubit coherence time^14^.

Despite the very simple structure of the Hamiltonian of equation 1, the calculation of the nuclear spin bath dynamics in a crystal is a very difficult task because of the many-body nature of the problem (each nuclear spin interacts with all other spins). When arbitrary inhomogeneous QI is added, the problem becomes unsolvable in practice. However, for the limiting cases of very small and very large QI, the nuclear spin echo decay times can be calculated with ~25% accuracy from first principles using the method of moments^16^. The details of the calculation techniques are discussed in the Methods and further in Supplementary Note 3; in what follows, we present the results of these calculations and use them to analyse the experimental data.

When quadrupolar shifts are much smaller than the dipolar interaction 
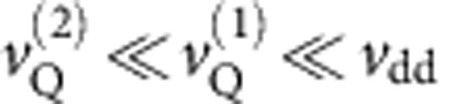
 (unstrained structures), the nuclear flip-flops are not affected by QI. The *T*_2_ times calculated for that case are shown in Fig. 4 with dashed lines for different isotopes. These calculated values are in good agreement with experiments on lattice-matched GaAs/AlGaAs structures, confirming the validity of the model employed. We note that the same *T*_2_ estimates are also valid for homogeneously strained structures^16^,^23^ (that is, where 
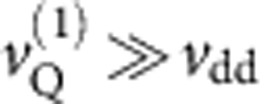
, but 
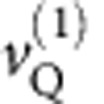
 is spatially homogeneous).

In the opposite case of very strong 
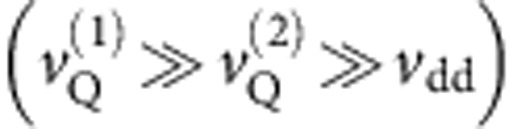
 and inhomogeneous QI, the dipole–dipole flip-flops become energetically forbidden. This effect can be described by truncating the off-diagonal flip-flop term in equation 1, leaving only the diagonal *Î*_z_*Ĵ*_z_ term in the Hamiltonian. However, even for completely suppressed flip-flops, the nuclear spin-echo coherence time (denoted as *T*_2,zz_) remains finite: the *Î*_z_*Ĵ*_z_ term still causes the nuclear spin decoherence (an effect known as instantaneous diffusion^30^). The *T*_2,zz_ sets an upper limit on the echo decay time *T*_2_. The *T*_2,zz_ times calculated for the studied InGaAs dots^16^ are shown by the solid lines in Fig. 4.

We now turn to the question of how nuclear spin *T*_2_ measurements can be used to predict the effect of the nuclear spin bath fluctuations on electron spin coherence. The electron spin decoherence is caused solely by the nuclear flip-flops^9^,^10^,^11^,^12^—the diagonal nuclear–nuclear interaction *Î*_z_*Ĵ*_z_ has no effect on the electron. If the flip-flops were completely suppressed, the electron spin would experience only a static nuclear field, which cannot cause any irreversible electron spin decoherence. Thus, in order to predict the electron spin coherence, we need to determine the nuclear flip-flop rates. As explained above, the experimental nuclear spin-echo *T*_2_ is controlled by both the nuclear flip-flops and the diagonal *Î*_z_*Ĵ*_z_ term of equation 1. To exclude the contribution of the diagonal interaction *Î*_z_*Ĵ*_z_, we examine how close the experimental *T*_2_ value is to the calculated limit *T*_2,zz_. For that, we introduce a characteristic nuclear spin flip-flop time *T*_2,ff_ defined as


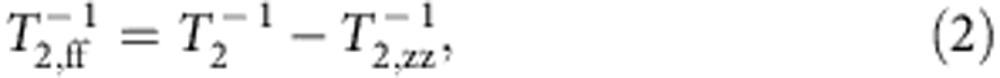


so that in an ideal QD with *T*_2,ff_ →∞ we would expect infinite electron spin coherence times.

It can be seen in Fig. 4 that *T*_2_<*T*_2,zz_ for In and both Ga isotopes implying only a partial suppression of the nuclear spin flip-flops. Using equation 2, we calculate *T*_2,ff_~5 ms for all three of those isotopes at low fields (1≲*B*_z_≲2 T). This *T*_2,ff_ is approximately three to eight times larger than it would have been in InGaAs/GaAs structures without inhomogeneous strain (*T*_2,ff_ values for this case are calculated to be *T*_2,ff_≈0.6, 1.5, 1.1 ms for ^115^In, ^69^Ga and ^71^Ga, see details in Supplementary Table 1 and Supplementary Note 3). We also note that the *T*_2_ of ^71^Ga and ^115^In decreases with increasing *B*_z_ in agreement with the fact that second-order quadrupolar shifts depend on magnetic field as 
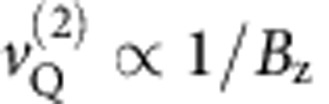
 (ref. 23), so that large *B*_z_ re-enables the nuclear flip-flops between the *I*_z_=±1/2 spin states. By contrast, no significant trend is observed for ^69^Ga most likely due to its smaller gyromagnetic ratio and larger quadrupolar moment making *T*_2_ less dependent on *B*_z_.

A very different picture is observed in Fig. 4 for arsenic nuclei: the values of *T*_2_ measured at *B*_z_=2–8 T coincide with the calculated *T*_2,zz_ within the experimental error. The value of *T*_2,ff_ calculated according to equation 2 in that case diverges becoming infinitely large: we can conclude that *T*_2,ff_>>5 ms for arsenic, implying very strong flip-flop suppression. These findings are consistent with the spectroscopic data in Fig. 1b, where the CT spectra of ^75^As are found to be ~10 times broader than for gallium nuclei, revealing much larger second-order quadrupolar shifts of arsenic nuclei, which are responsible for the strong suppression of the nuclear spin exchange flip-flops.

## Discussion

Second-order quadrupolar shifts 
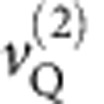
 appear whenever *V*_ij_ is not a cylindrically symmetric tensor with its main axis along *B*_z_ (ref. 23). One obvious reason for low-symmetry *V*_ij_ in self-assembled QDs is non-uniaxial symmetry of the elastic strain tensor, or deviation of the strain main axis from *B*_z_. Such a mechanism is likely to be the main cause of the CT inhomogeneous broadening of gallium and indium, resulting in the above increase in *T*_2,ff_ by a factor of ~3–8.

For the anion ^75^As, the picture is different: the additional second-order shifts 
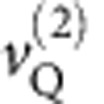
 are induced by random alloy mixing of the cationic Ga and In atoms. Each arsenic nucleus has four nearest neighbours, and unless all of them are of the same type (all gallium or all indium) a non-zero *V*_ij_ will appear^18^. Furthermore, unlike the elastic strain fields that change gradually over many crystal unit cells, the configuration of the neighbouring atoms is random, so that even the nearest arsenic nuclei (which have the strongest dipolar coupling) can have very different 
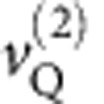
. Such compositional disorder induces large spatially inhomogeneous CT frequency shifts, drastically suppressing the flip-flops. We propose that such effects can be used to engineer QDs with a frozen nuclear spin bath. One possible approach is to substitute some of the arsenic nuclei with antimony and/or phosphorus: in such InGaAsSb(P) QDs gallium and indium spins will also experience large inhomogeneous 
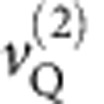
 shifts because of atomic-scale alloy disorder, resulting in an overall slow down of the flip-flops for all isotopes.

The increase of the nuclear spin-echo *T*_2_ observed for the CTs is driven by the second-order quadrupolar shifts 
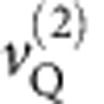
, which are significantly smaller than the first-order shifts 
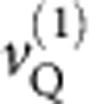
 (
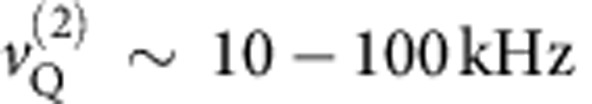
 compared with 
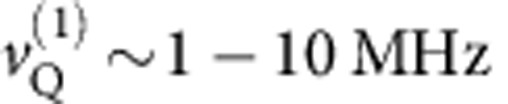
). Therefore, we conclude that the flip-flops of the nuclei in |*I*_z_|>1/2 states (affected by 
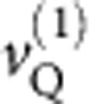
) are effectively frozen for all isotopes in strained self-assembled InGaAs dots. Consequently, electron spin decoherence in self-assembled dots is caused solely by the flip-flops of the nuclei in *I*_z_=±1/2 states.

As we have shown the nuclear *T*_2_ time increases when magnetic field is reduced down to *B*_z_=2 T. We expect this trend to continue down to magnetic fields where the nuclear Zeeman frequency *ν*_Z_ becomes comparable to the first-order quadrupolar shifts 
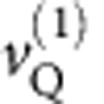
. For ^75^As, we have 
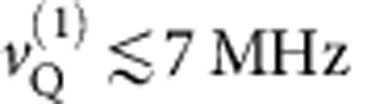
 and *ν*_Z_/*B*_z_≈7.33 MHz T^−1^, whereas other isotopes have even larger *ν*_Z_/*B*_z_ and smaller 
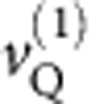
. Thus, our results are expected to be valid at least down to *B*_z_≈1 T. At lower magnetic fields, the nuclear spin energy levels cross^31^, which may significantly alter the nuclear spin flip-flop dynamics—this regime requires further experimental investigation.

Finding an exact relation between the nuclear spin bath coherence times and the central spin coherence times is a complicated problem, in particular when QIs are involved^15^. However, some general qualitative conclusions can be readily drawn. First, it has been shown that for QDs the spectral diffusion is well into the ‘slow bath’ regime (as opposed to ‘motional narrowing’)^10^, thus the extended nuclear spin coherence times reported here are expected to result in longer central spin coherence times. Second, dynamic nuclear spin polarization^32^, which enhances the occupancy of the |*I*_z_|≈*I* states can be used to effectively depopulate and ‘dilute’ the *I*_z_=±1/2 states resulting in further reduction of the nuclear spin fluctuations. Recently, electron spin coherence times >200 μs in lattice-matched electrostatic GaAs/AlGaAs QDs^1^ were observed. The self-assembled InGaAs/GaAs dots typically have a factor of ~30 fewer nuclear spins and hence a factor of ~5 stronger fluctuations. This is compensated by a factor of ~5 slow-down of nuclear flip-flops observed in this work. Thus, we expect that electron coherence times of the similar scale (~100 μs) could be achieved in self-assembled dots, if other sources of decoherence such as charge noise and interaction with phonons^5^,^6^ can be eliminated.

In conclusion, we have demonstrated the first direct probing of the coherent nuclear spin bath dynamics in inhomogeneously strained QDs. We anticipate that electron(hole) spin qubits in self-assembled structures exhibiting large inhomogeneous QIs^14^,^15^ have a significant advantage over the lattice-matched counterparts^1^. As an outlook, we note that pulsed NMR techniques employed here to study direct nuclear–nuclear interactions in neutral dots can be readily applied to charged dots. This will provide insight into coherent nuclear spin dynamics in the presence of the Knight field^33^ and indirect electron(hole)-mediated nuclear–nuclear spin interactions, which were previously shown to be significant for longitudinal nuclear spin relaxation (with *T*_1_~100 s)^34^. Furthermore, our NMR techniques are not restricted to spin-echo and can be easily extended to accommodate the whole variety of pulse sequences used in Fourier transform NMR, offering a powerful tool to explore the many-body physics of interacting nuclear spins in strained nanostructures.

## Methods

### QD sample structure

The InGaAs/GaAs sample consists of a single layer of nominally InAs QDs placed within a microcavity structure, which is used to select and enhance the photoluminescence from part of the inhomogeneous distribution of QD energies. The sample was grown by molecular beam epitaxy. The QDs were formed by deposition of 1.85 monolayers of InAs—just above that required for the nucleation of dots. As a result, we obtain a low density of QDs at the post-nucleation stage. The cavity is formed between an asymmetric set of distributed Bragg reflector pairs, which uses 16 pairs of GaAs/Al_0.8_Ga_0.2_As below and 6 pairs above the cavity. The cavity *Q* factor is ~250 and the cavity has a low temperature resonant wavelength at around 920 nm. The luminescence of the QDs is further enhanced by a short-period GaAs/AlAs superlattice surrounding the QD layer.

### Continuous wave NMR spectroscopy

The CT spectra of an individual InGaAs/GaAs QD shown in Fig. 1b were measured using inverse method which provides >8 times CT signal enhancement for *I*=3/2 nuclei^13^. The NMR signal is calculated as the hyperfine shift of the QD Zeeman doublet divided by the spectral gap width, so that the values on the vertical scale give the spectral density of the distribution of the nuclear resonance frequencies. The spectral gap width (determining the spectral resolution) is 6 kHz for the ^69^Ga spectra, and 16 kHz (32 kHz) for the *B*_z_=8 T (*B*_z_=2 T) spectrum of ^75^As. For convenience, the spectra are plotted as a function of Δ*ν*=*ν*−*ν*_Z_, where *ν*_Z_ is a constant proportional to the isotope gyromagnetic ratio: *ν*_Z_/*B*_z_≈7.33 MHz T^−1^ for ^75^As and *ν*_Z_/*B*_z_≈10.3 MHz T^−1^ for ^69^Ga.

### Techniques for pulsed NMR measurements

We implement optically detected pulsed NMR techniques, which extend the techniques and are based on the results of our previous work of ref. 13. The timing diagram of one measurement cycle and the changes to nuclear spin polarization are shown schematically in Fig. 2 (spin *I*=3/2 is used as an example). The cycle starts with optical nuclear spin pumping^22^,^32^ using high-power *σ*^+^ circularly polarized laser [stage (a)]. Spin polarized electrons of highly excited and/or multiexcitonic states transfer their polarization to nuclear spins via the hyperfine interaction^35^. The pump duration is chosen long enough (~3–7 s depending on magnetic field *B*_z_) to achieve the steady-state nuclear spin polarization. Nuclear polarization degrees exceeding 50% are obtained, which means that a large portion of the nuclei is initialized into the *I*_z_=−3/2 state. To make the NMR signal of the CT detectable, the population of the *I*_z_=−1/2(+1/2) state must be maximized (minimized). This is done at stage (b) using population transfer technique^36^: an rf field containing two frequency components is applied, the frequencies are swept over both satellite transition bands −3/2↔−1/2 and +1/2↔+3/2 resulting in adiabatic inversion of the populations of the −3/2 and −1/2 states as well as +1/2 and +3/2 states. Following that a sequence of rf pulses resonant with the CT is applied (stage (c)). Different sequences can be implemented, depending on the experiment: a single pulse of a variable duration is used for Rabi-oscillation measurements (Fig. 3a), whereas a three-pulse sequence is used to measure either the spin-echo (Fig. 3b, 90°−*τ*_0_−180°−*τ*−90° sequence with *τ*_0_ fixed to 0.4 ms) or spin-echo decay (Fig. 3c, 90°−*τ*−180°−*τ*−90° sequence). The rf amplitude is chosen to give 90° phase rotation for 3- to 8-μs-long pulses (depending on isotope). This corresponds to pulse bandwidths of ~100 kHz, and, as satellite transitions are shifted by the much bigger (~1–10 MHz) first-order quadrupolar shifts, this ensures selective excitation of the CT. Finally (stage (d)), we probe the effect of the NMR pulse sequence by measuring the changes in the average nuclear spin polarization ‹*I*_z_› on the single QD. This is achieved by exciting the dot with a short (~1–4 ms depending on *B*_z_) probe laser pulse and measuring the hyperfine shifts in the QD photoluminescence spectrum^13^,^22^,^32^. To improve the signal-to-noise ratio, the experimental cycle is repeated 20–50 times for each parameter value (for example, for each value of 2*τ* in Fig. 3c). Further details of experimental techniques can be found in Supplementary Note 1.

### Theoretical model

Nuclear spin decoherence is a result of nuclear–nuclear spin interactions: each individual nuclear spin has its own spin environment producing additional magnetic field, which changes the resonant frequency of that nucleus. Thus, the problem of calculating the nuclear spin decoherence is equivalent to the problem of calculating homogeneous NMR line broadening. In principle, this problem can be solved by diagonalizing the Hamiltonian of the nuclear–nuclear interactions. This, however, is practically impossible even for a system of few tens of spins, let alone the whole crystal. An insightful solution to this difficulty has been found by Van Vleck^17^,^23^, who showed that the moments of the NMR lineshape can be expressed as traces of certain quantum mechanical operators. The key property of the trace is that it can be calculated in any wavefunction basis, hence diagonalization of the Hamiltonian is not needed. This technique does not allow an exact resonance lineshape to be calculated, but in most cases the second moment *M*_2_ (corresponding to the homogeneous NMR linewidth) contains sufficient information.

The nuclear spin coherence time can be estimated as 
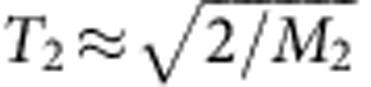
. The calculation of *M*_2_ for a whole crystal is a straightforward but very tedious process involving summation of various matrix elements. If one wants to calculate the spin-echo coherence time (as opposed to the free-induction decoherence time), some of the matrix elements must be discarded from the summation. QI is also taken into account by further truncation of the sums. The details of these calculations can be found in refs 16, 17, 23 and are also outlined in Supplementary Note 3.

## Author contributions

M.H. developed and grew the samples. E.A.C. developed the techniques, carried out the experiments and analysed the data. E.A.C., M.S.S. and A.I.T. wrote the manuscript.

## Additional information

**How to cite this article:** Chekhovich, E. A. *et al.* Suppression of nuclear spin bath fluctuations in self-assembled quantum dots induced by inhomogeneous strain. *Nat. Commun.* 6:6348 doi: 10.1038/ncomms7348 (2015).

## Supplementary Material

Supplementary Information Supplementary Figures 1-3, Supplementary Table 1, Supplementary Notes 1-3, and Supplementary References
