# Supplementary material for: Suppression of nuclear spin bath fluctuations in self-assembled quantum dots induced by inhomogeneous strain
Source: Nat Commun. 2015 Feb 23;6:6348. doi: 10.1038/ncomms7348 (PMC4346613; doi:10.1038/ncomms7348)
Supplement: Supplementary Information — Supplementary Figures 1-3, Supplementary Table 1, Supplementary Notes 1-3, and Supplementary References [file ncomms7348-s1.pdf]

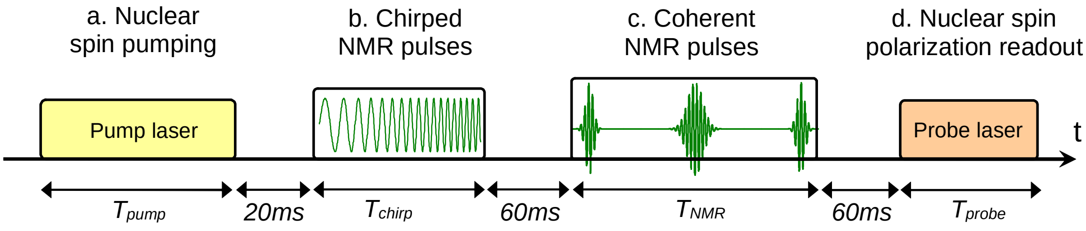

Supplementary Figure 1. **Timing diagram for pulsed NMR experiments on strained quantum dots.** See detailed explanation in Supplementary Note 1 A.

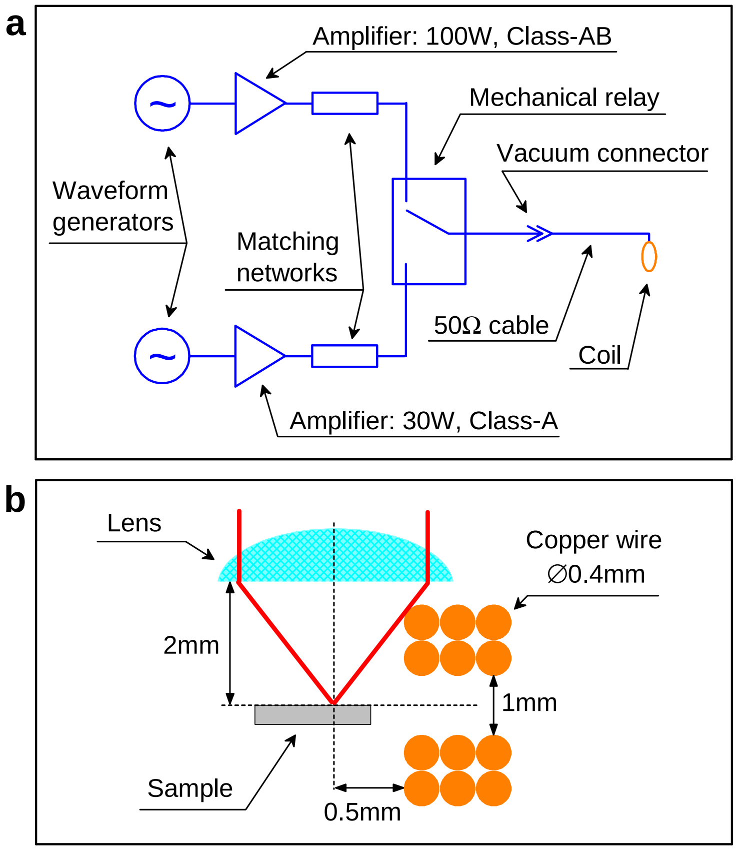

Supplementary Figure 2. **Hardware implementation of the pulsed NMR techniques. a,** Circuit diagram. **b,** Schematic drawing of the NMR coil arrangement (side view). See detailed explanation in Supplementary Note 1 B.

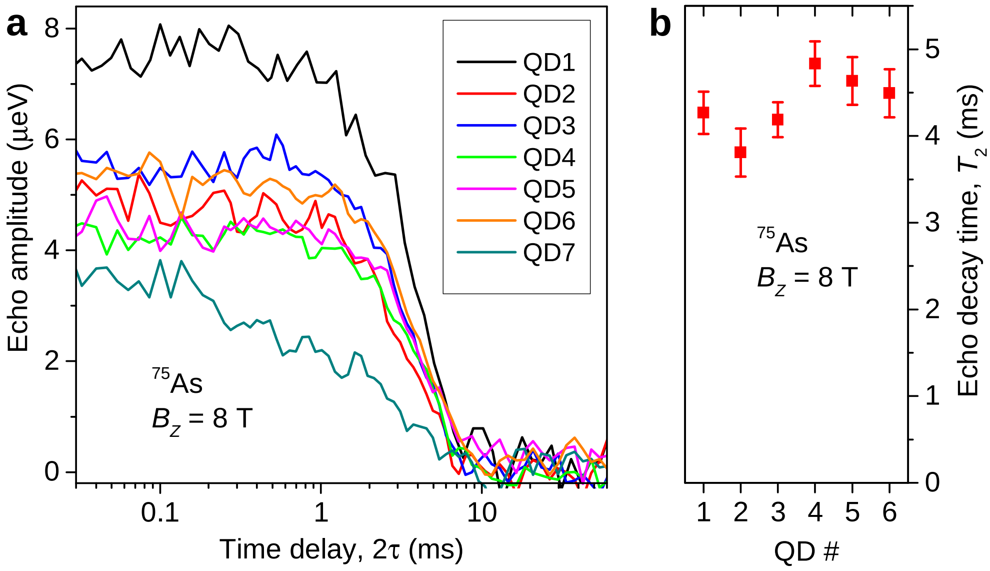

Supplementary Figure 3. **Additional echo decay measurements.** **a,** Spin echo decay curves of ^{75}As measured for dots QD1-QD7 at $B_z = 8$ T. **b,** Spin echo decay times T_2 obtained for dots QD1-QD6 using Gaussian fitting of the curves in **a** (90% confidence intervals).

TABLE Supplementary Table 1. Nuclear spin parameters and calculated nuclear spin echo decay times T_2

Parameter	^{75}As	^{115}In	^{69}Ga	^{71}Ga
Nuclear spin I	3/2	9/2	3/2	3/2
Gyromagnetic ratio γ (10^7 s^{-1}/T)	4.596	5.897	6.439	8.181
Natural abundance	1	0.957	0.601	0.399
Abundance in studied quantum dots ρ	1	0.23	0.46	0.3
Calculated T_2 times (ms) for the studied dots:				
Strongly inhomogeneous quadrupolar shifts. $T_{2,\mathrm{zz}}$	4.03	8.08	3.04	2.31
Homogeneous quadrupolar shifts. $T_{2,\mathrm{zz+ff}}$	1.3	0.55	0.98	0.75
Homogeneous quadrupolar shifts, including the effect of heteronuclear couplings. $T_{2,\mathrm{zz+ff+IS}}$	0.93	0.42	0.66	0.51

Supplementary Note 1. DETAILS OF PULSED NMR TECHNIQUES

We implement optically detected pulsed NMR techniques which extend the techniques of our previous work[2,3]. Some description of experimental techniques was previously reported in the Supplementary of Ref.[2] and also applies to this work. Below we focus on the techniques specific to the present work.

A. Pump-probe techniques for optically detected pulsed NMR

The timing diagram of one measurement cycle is shown schematically in Supplementary Fig. 1 (this is a detailed version of diagram in Fig. 2 of the main text). The cycle consists of the following four stages:

Stage a. In order to achieve sufficiently large NMR signal the nuclei must be prepared in a highly-polarized state. Manipulation of nuclear spin polarization relies on the hyperfine interaction of electrons and nuclear spins. Excitation with a σ^+ circularly polarized "pump" lazer generates spin polarized electrons, which transfer their polarization to nuclear spins via the hyperfine interaction[4–9]. In this work we use high power nonresonant optical pumping so that large dynamic nuclear spin polarization (polarization degrees exceeding 50%) is induced via hyperfine interaction with highly-excited and/or multiexcitonic quantum dot states[1]. The pumping wavelength of ∼850 nm corresponds to excitation into the QD wetting layer states. Optical powers exceeding the QD saturation level by more than a factor of 10 are typically used[1,9]. At these powers the dependence of the steady-state nuclear polarization on the optical power saturates. This ensures large nuclear spin polarization degree as well as its good reproducibility due to insensitivity to laser power fluctuations. Pump durations of $T_{\rm pump} = 3 - 7$ s (depending on magnetic field $B_{\rm z}$) are used to achieve a steady-state polarization independent of the polarization left after the previous cycle. A delay of 20 ms is introduced after the pumping to ensure that the pump laser is completely blocked by a mechanical shutter, so that NMR on a quantum dot is measured in the dark.

Stage b. The amplitude of the NMR signal of the central transition (CT) studied in this work is determined by the difference in population probabilities of the $I_{\rm z} = -1/2$ and $I_{\rm z} = +1/2$ nuclear spin states. However, for spin $I > 1/2$ inducing large nuclear spin polarization degree does not necessarily increase the CT signal. In fact, in the limit of 100% polarization all nuclei will be in the $I_{\rm z} = -I$ state and the NMR signal of CT will vanish. Simple analysis shows that any optically induced distribution would yield CT NMR signal too weak to detect in our setup. Thus nuclear

spin level populations have to be manipulated artificially. We achieve this via "population transfer" techniques[10] which uses chirped radio-frequency (rf) pulses. For spin-3/2 nuclei we use an rf field containing two spectral components. The frequencies of both components are swept "outwards" from $\nu_{\mathrm{CT}} \pm \nu_{\mathrm{i}}$ to $\nu_{\mathrm{CT}} \pm \nu_{\mathrm{f}}$, where ν_{CT} is the frequency of the central transition. The initial frequency offset is chosen to be $\nu_{\mathrm{i}} = 20$ kHz, whereas the final offset is chosen to be larger than the maximum first-order quadrupolar shift of the studied isotope ($\nu_{\mathrm{f}} = 8.5$ MHz for ^{75}As, $\nu_{\mathrm{f}} = 5.6$ MHz for ^{69}Ga and $\nu_{\mathrm{f}} = 3.5$ MHz for ^{71}Ga are used). Such frequency sweeps adiabatically swap the populations of the $-3/2$ and $-1/2$ states as well as $+1/2$ and $+3/2$ states, significantly enhancing the population difference of the $\pm 1/2$ states. For spin-9/2 indium the sweeps are done "inwards" ($\nu_{\mathrm{i}} > \nu_{\mathrm{f}}$), with $\nu_{\mathrm{i}} = 8.5$ MHz and $\nu_{\mathrm{f}} = 120 - 230$ kHz, which effectively transfers the populations of the $\pm 9/2$ states to the $\pm 1/2$ states. The amplitude of each swept component is $\sim 0.2 - 0.5$ mT. The sweep rate is calibrated in an additional measurement and is chosen to maximize the CT signal. Typical rates used are $\sim 7 - 20$ MHz/s, so that the duration of the chirped pulse is $T_{\mathrm{chirp}} \sim 0.3 - 1.5$ s. After the sweep a 60 ms delay is introduced to permit any transverse nuclear magnetization to decay and allow a mechanical relay to switch between the "chirp" and "pulse" rf signal sources (see Supplementary Note 1 B).

Stage c. A sequence of rf pulses resonant with the central transition is applied to manipulate coherently the magnetization of the $I_{\mathrm{z}} = \pm 1/2$ nuclear spin subspace. Different sequences can be implemented, e.g. Rabi-oscillations, Hahn-echo or echo decay, as demonstrated in Fig. 3 of the main text. The rf amplitude is chosen to give 90° phase rotation of the $I_{\mathrm{z}} = \pm 1/2$ subspace for 3-8 μs long pulse (depending on isotope). All pulse sequences are designed in a way that the final magnetization that needs to be measured is projected on to the Oz axis, so that it can be detected optically. For example in the echo decay sequence $90° - \tau - 180° - \tau - 90°$ the last 90° pulse rotates the transverse magnetization (which is of interest) aligning it along the Oz axis. The total duration of the NMR pulse sequence T_{NMR} varies from a few microseconds to 110 ms. After the pulse sequence a 60 ms delay is introduced to allow the decay of any spurious transverse nuclear magnetization and the dissipation of heating induced by chirped or resonant NMR pulses.

Stage d. Finally we probe the effect of the NMR pulse sequence by measuring the changes in the average nuclear spin polarization $\langle I_{\mathrm{z}} \rangle$ on the dot. This is achieved by exciting the dot with a short ($T_{\mathrm{probe}} = 1 - 4$ ms depending on B_{z}) nonresonant (~ 850 nm) linearly polarized probe laser pulse and measuring the hyperfine shifts of the Zeeman splitting in the QD photoluminescence spectrum[2–4]. The power of the probe laser is $\sim 1/10$ of the QD saturation power. For each magnetic field we perform an additional measurement where we select T_{probe} for which the parasitic changes

induced by the probe pulse do not exceed $\sim 2\%$ of the initial nuclear spin polarization[3]. We use differential measurements: for spin-echo and echo decay experiments the NMR signal is calculated as the difference of the QD Zeeman splitting measured with the $90^\circ - \tau - 180^\circ - \tau - 90^\circ$ sequence and the splitting measured with the $90^\circ - 100$ ms-180° sequence. In this way a complete decay of echo corresponds to ~ 0 μeV signal. For Rabi-oscillations measurements we subtract the Zeeman splitting obtained from a measurement with a single 90° pulse.

The total duration of stages $b - d$ does not exceed ~ 2 s, which is much shorter than the nuclear spin polarization longitudinal (T_1) decay time (>1 hour). In order to improve the signal to noise ratio the experimental cycle is repeated $10 - 50$ times during the photoluminescence spectrum acquisition for each parameter value [e.g. for each value of 2τ in the spin echo measurements].

B. Pulsed optically detected NMR: the hardware

A schematic circuit diagram of the NMR setup is shown in Supplementary Fig. 2(a). The "chirp" and the "resonant" pulses are generated in separate arms, each containing a digital arbitrary/function generator, a high power amplifier and a matching network. The signal from one of the two arms is selected by a mechanical relay and is then transmitted into the low temperature vacuum insert via a coaxial cable. All equipment and cables have 50 Ω impedance, while the coil impedance differs significantly from 50 Ω, hence the need for the matching networks. For resonant NMR pulses only a narrow bandwidth is required (few hundred kHz); thus we use a single stub network which gives nearly ideal impedance matching at a specific frequency. The "chirped" pulse requires a much larger bandwidth (up to 20 MHz), over which it is impossible to achieve good impedance matching. In that case the role of the matching network (consisting of lumped LC elements and cables of different length) is to provide nearly constant transmission over the frequency sweep band. The large mismatch of such a network is compensated by the use of a mismatch-tolerant class-A power amplifier.

A schematic drawing of the NMR coil arrangement is shown in Supplementary Fig. 2(b). The coil consists of 6 turns of a copper wire (diameter 0.4 mm) wound in two layers. The external radius of the coil is smaller than the working distance of the lens used to excite and collect photoluminescence. As a result the coil can be positioned very close to the edge of the quantum dot sample: the distance between the coil edge and the lens focal point is ∼0.5 mm. The lens and the coil are fixed while the sample is mounted on an XYZ piezo-positioner allowing the sample surface to be scanned and different individual dots to be studied. For the range of frequencies used (5-110

MHz) the coil produces an oscillating magnetic field ∼20 mT for an input power of 100 W.

Supplementary Note 2. ADDITIONAL EXPERIMENTAL RESULTS

All experimental results presented in the main text were measured on the same quantum dot QD1. To verify the consistency of our conclusions we have carried out spin echo decay measurements on an additional set of different dots (QD2-QD7) from the same sample. Spin echo decay curves of ^{75}As measured for dots QD1-QD7 at $B_z = 8$ T are shown in Supplementary Fig. 3(a) with different colours.

Quantum dots QD1-QD6 demonstrate echo decay that can be well described by a Gaussian function. By contrast, one of the dots (QD7) demonstrates significantly faster non-Gaussian decay.

The echo decay times T_2 obtained from Gaussian fitting are shown in Supplementary Fig. 3(b) for QD1-QD6. There is some dot-to-dot variation. However, it is comparable to the experimental error, and all of the experimental T_2 values are also in good agreement with the calculated decay time of ∼4.03 ms, confirming the reproducibility of the presented experimental results and analysis.

The considerably faster echo decay observed for QD7 is not fully understood. One possibility is that additional nuclear spin decoherence is caused by charge fluctuations. Such fluctuations can not be controlled in our experemints, since we are using electric-gate-free structures. Thus electrons or holes can hop between the nearby impurities or can randomly populate the dot. This might also be the cause for some dot-to-dot variations in T_2 values and will be a subject of further studies (e.g. using dots in Schottky-diode structures).

Supplementary Note 3. FIRST PRINCIPLE CALCULATION OF THE NUCLEAR SPIN-ECHO DECAY TIME T_2

Our calculations are done within a framework developed by Haase and Oldfield in Ref.[11]. Their model is based on the well-known method of moments initially developed by Van Vleck[12]. Below we outline the key points of this model and show how it is used to derive the results presented in the main text.

The decay of the nuclear spin echo is caused by the dipole-dipole interaction between nuclear spins. At sufficiently large magnetic field along Oz (above a few mT) the interaction between two

nuclear spins I and J is described by the "truncated" dipole-dipole Hamiltonian[13]:

$$\hat{H}_{\rm dd}/(2\pi\hbar) = \nu_{\rm dd}\left[\hat{I}_{\rm z}\hat{J}_{\rm z} - \frac{1}{2}(\hat{I}_{\rm x}\hat{J}_{\rm x} + \hat{I}_{\rm y}\hat{J}_{\rm y})\right],$$
$$\nu_{\rm dd} = \frac{\mu_0}{4\pi}\frac{\hbar}{2\pi}\gamma_{\rm I}\gamma_{\rm J}\frac{1-3\cos^2\theta}{r^3}, \quad (1)$$

where $\hat{I}$ and $\hat{J}$ are the nuclear spin operators, and $\nu_{\rm dd}$ is the coupling strength. $\nu_{\rm dd}$ depends on the gyromagnetic ratio $\gamma_{\rm I}$ ($\gamma_{\rm J}$) of the spin $I(J)$, the internuclear distance r and the angle θ between the magnetic field and the vector $\mathbf{r}$ connecting the nuclei ($\nu_{\rm dd} \lesssim 200$ Hz in frequency units for all nuclei in InGaAs sample).

The interactions between nuclei of the same type (homonuclear coupling) and the nuclei of different types (heteronuclear coupling) have a different role in nuclear spin echo decay. The dephasing caused by the homonuclear coupling is not refocused by the 180° pulse and, as a result, the spin echo decays. The heteronuclear coupling between the "studied" spins of isotope I and the spins of "another" isotope S, can be viewed as a randomly distributed magnetic field induced by isotope S and acting on I. On sufficiently short time-scales this effective field is static and thus its effect is refocused by the 180° pulse. However, the spin flip-flops induced by the homonuclear coupling of the S nuclei will make this effective magnetic field time-dependent. As a result the spin echo of isotope I will decay via the spectral diffusion mechanism (this is exactly the same mechanism that causes the decoherence of the electron spin interacting with the nuclear spin bath[14–16]). Below we will consider both homonuclear and heteronuclear interaction.

Quadrupolar interactions modify nuclear spin spectra and affect nuclear spin dephasing dynamics (for introduction to quadrupolar effects see Chapter X of Ref.[17]). An exact treatment of nuclear spin echo decay under the combined effect of quadrupolar and dipole-dipole Hamiltonians is unattainable. However, the echo decay times can be obtained relatively easily for two limiting cases[11]: (i) The quadrupolar interaction is strongly inhomogeneous, i.e. the difference in quadrupolar shifts (including the second-order shifts) of the nearby nuclei is much larger than their dipolar coupling. Under these conditions all dipolar nuclear flip-flops are energetically forbidden. Such a case would correspond to a self-assembled quantum dot where first-order quadrupolar shifts are strongly inhomogeneous due to inhomogeneous elastic strain, while second-order shifts are inhomogeneous due to atomic-scale disorder. (ii) The opposite case is observed for homogeneous quadrupolar interactions, i.e. each nucleus of a particular isotope is subject to the same electric field gradient. In that case the flip-flops between nuclear spins I and J of the type $(I_{\rm z} = m, J_{\rm z} = m \pm 1) \leftrightarrow (I_{\rm z} = m \pm 1, J_{\rm z} = m)$ are energetically allowed. This case is relevant for uniformly strained GaAs/AlGaAs quantum wells[18–20]. We now consider these two cases

in detail.

A. Nuclear spin echo decay under strongly inhomogeneous quadrupolar shifts [case (i)].

This case is the easiest for numerical calculations. Since all flip-flops are forbidden, the only source of nuclear spin echo decay is due to the diagonal ($\hat{I}_{\rm z}\hat{J}_{\rm z}$) part of the homonuclear dipole-dipole interaction (heteronuclear coupling has no effect since there are no flip-flops that can cause spectral diffusion).

The nuclear spin echo decay time T_2 (the time during which the echo amplitude decays to $1/e$ of its initial value) due to the homonuclear coupling can be approximated as[11]:

$$T_2 = \sqrt{2/M_{\rm 2E}}, \tag{2}$$

where $M_{\rm 2E}$ is the second moment of the dipole-dipole interaction (Supplementary Eq. 1) calculated for the whole crystal:

$$M_{\rm 2E} = F\left(\frac{\mu_0}{4\pi}\right)^2 \hbar^2\gamma^4 a^{-6}\rho\sum_{\rm i\neq 0}\left(\frac{3}{2}\times\frac{1-3\cos^2\theta_{\rm i}}{(r_{\rm i}/a)^3}\right)^2, \tag{3}$$

where μ_0 is vacuum magnetic permeability (introduced here to carry out calculations in SI units), $\hbar$ - Planck's constant, γ - nuclear gyromagnetic ratio. The summation goes over the whole crystal, $r_{\rm i}$ is the length of the vector $\mathbf{r}_{\rm i}$ between the i-th nucleus and the nucleus chosen as an origin (i=0), $\theta_{\rm i}$ is the angle between $\mathbf{r}_{\rm i}$ and the direction of magnetic field, a - is the lattice constant. ρ - is the isotope abundance. Here we take into account that all isotopes are sufficiently abundant ($\rho > 0.1$) so that $M_{\rm 2E} \propto \rho$ (Chapter IV, §9 of Ref.[13]). The factor F depends on the nature of the quadrupolar shifts and the studied transition. For spin-echo decay of the central transition under selective excitation and under strongly inhomogeneous quadrupolar shifts [case (i)] it is:

$$F = \frac{2}{9(2I+1)}, \tag{4}$$

The fcc lattice sum is calculated numerically

$$\sum_{\rm i\neq 0}\left(\frac{3}{2}\times\frac{1-3\cos^2\theta_{\rm i}}{(r_{\rm i}/a)^3}\right)^2 \approx 155.13, \tag{5}$$

Since ^{75}As is the only isotope of arsenic, we have $\rho = 1$. The relative concentration of indium and gallium in the studied dots is estimated to be 0.24 and 0.76 respectively[2]. Taking also into account the natural abundances we have $\rho \approx 0.46$ for ^{69}Ga, $\rho \approx 0.30$ for ^{71}Ga and $\rho \approx 0.23$ for ^{115}In. These and other nuclear parameters are listed in Supplementary Table 1.

The low temperature lattice constant of GaAs is $a_0 = 0.564786$ nm. The lattice constant of bulk InAs is larger by 7.17%. To take into account that only 24% of atoms are indium we use linear interpolation of lattice constant dependence on indium concentration. Furthermore the lattice constant is modified by the elastic strain. To estimate this effect we first need to imagine that an InGaAs dot is uniformly compressed to match the lattice constant of GaAs and is embedded into the unstrained GaAs matrix in a defect-free manner[21]. Such structure is not in equilibrium - the strain fields have to relax resulting in quantum dot expansion. Theoretical analysis shows that a strained InGaAs dot recovers $\sim 2/3$ of the changes in size that were initially induced by compression[21]. Thus for the lattice constant we use the following estimate $a \approx a_0(1+\frac{2}{3}\times 0.24\times 0.0717) \approx 0.571$ nm.

Using these values we calculate the following echo decay times for case (i) which we denote as $T_{2,\mathrm{zz}}$: 4.03 ms for ^{75}As, 3.04 ms for ^{69}Ga, 2.31 ms for ^{71}Ga and 8.08 ms for ^{115}In. These $T_{2,\mathrm{zz}}$ values are also given in Supplementary Table 1 and are shown by the solid lines in Fig. 4 of the main text.

B. Nuclear spin echo decay under homogeneous quadrupolar shifts [case (ii)].

Nuclear spin echo decay in case (ii) is caused by both homonuclear and heteronuclear dipole-dipole couplings. First we consider the homonuclear interaction: now in addition to the diagonal part ($\propto \hat{I}_{\mathrm{z}}\hat{J}_{\mathrm{z}}$), the flip-flop part ($\propto \hat{I}_{\mathrm{x}}\hat{J}_{\mathrm{x}} + \hat{I}_{\mathrm{y}}\hat{J}_{\mathrm{y}}$) of the Supplementary Eq. 1 also contributes to echo decay. This is taken into account by using a different value of F in Supplementary Eq. 3 which now reads[11]:

$$F = \frac{2}{9(2I+1)} \times \frac{80I^4 + 160I^3 + 344I^2 + 264I + 333}{256}. \quad (6)$$

The resulting echo decay times (which we denote as $T_{2,\mathrm{zz+ff}}$) are 1.3 ms for ^{75}As, 0.98 ms for ^{69}Ga, 0.75 ms for ^{71}Ga and 0.55 ms for ^{115}In (see also Supplementary Table 1).

It follows from Eqns. 2, 4, 6 that the ratio of homonuclear decay times in case (i) and case (ii) depends only on spin I: $T_{2,\mathrm{zz}}/T_{2,\mathrm{zz+ff}} \approx 3.1$ for $I = 3/2$ and $T_{2,\mathrm{zz}}/T_{2,\mathrm{zz+ff}} \approx 14.8$ for $I = 9/2$. We also note here that the factor F determining the echo decay of the central transition is most influenced by the inhomogeneity of the second-order quadrupolar shifts. By contrast the nature of the first-order quadrupolar shifts makes little difference: values of F differing by less than 10% from that of Supplementary Eq. 6 can be derived for situations when (a) quadrupolar effects are zero, (b) both first and second order quadrupolar effects are homogeneous, (c) first order effects are inhomogeneous, while second order effects are homogeneous[11,22]. Thus very similar echo decay

times are expected for bulk GaAs, uniformly strained and strain-free GaAs/AlGaAs quantum wells.

In order to characterize the timescales of the nuclear flip-flops we introduce the flip-flop time as (Eq. 2 of the main text):

$$T_{2,\mathrm{ff}} = 1/(T_2^{-1} - T_{2,\mathrm{zz}}^{-1}), \tag{7}$$

where T_2 is the experimentally measured echo decay time. When homonuclear flip-flops are strongly suppressed, $T_2 \to T_{2,\mathrm{zz}}$ according to the analysis for case (i), so that $T_{2,\mathrm{ff}} \to \infty$ according to Supplementary Eq. 7. To estimate the $T_{2,\mathrm{ff}}$ in the absence of inhomogeneous quadrupolar effects we use the same Supplementary Eq. 7 but with the calculated $T_{2,\mathrm{zz+ff}}$ instead of experimental T_2. The resulting $T_{2,\mathrm{ff}}$ values are 1.5 ms, 1.1 ms and 0.6 ms for ^{69}Ga, ^{71}Ga and ^{115}In respectively (these values are also quoted in the main text).

We now also include the effect of heteronuclear dipole-dipole interaction which causes additional echo decay via spectral diffusion. This effect can not be treated exactly, but it is possible to obtain a reasonable estimate.

First we estimate the root mean square amplitude of the effective field that the "fluctuating" isotope S exerts on the "studied" isotope I. For that we calculate the heteronuclear interaction second moment:

$$M_{2\mathrm{E,S}} = F\left(\frac{\mu_0}{4\pi}\right)^2 \hbar^2\gamma_\mathrm{I}^2\gamma_\mathrm{S}^2 a^{-6}\rho_\mathrm{S}\sum_{\mathrm{i}\neq 0}\left(\frac{3}{2}\times\frac{1-3\cos^2\theta_\mathrm{i}}{(r_\mathrm{i}/a)^3}\right)^2$$
$$F = \frac{2}{9(2S+1)}, \tag{8}$$

where I and S indices denote the values of the corresponding isotopes. In the lattice sum the summation now goes either over the same fcc sublattice (e.g. for interaction between In and Ga), which gives the same value as in Supplementary Eq. 5, or over the "other" sublattice (for interaction of As with In or Ga), which gives a lattice sum of ≈ 113.30.

The echo decay time of the I isotope will depend both on the coupling strength (characterized by $M_{2\mathrm{E,S}}$) and the correlation time τ of the flip-flops of the S nuclei. The fastest decoherence of the I nuclei will take place when $\tau \times \sqrt{M_{2\mathrm{E,S}}} \sim 1$. Using a Gaussian approximation[23] an upper bound on the decay rate under these conditions can be estimated as $\sim \sqrt{M_{2\mathrm{E,S}}}/1.62$, where the 1.62 factor is a root of a transcendental equation derived in Ref.[23] under the assumption of an exponential correlation function. The overall spin echo decay time of the I nuclei can then be calculated as

$$T_{2,\mathrm{zz+ff+IS}} = \left(T_{2,\mathrm{zz+ff}}^{-1} + \sum_{\mathrm{S}\neq\mathrm{I}}\sqrt{M_{2\mathrm{E,S}}}/1.62\right)^{-1}, \tag{9}$$

where the summation goes over all isotopes S distinct from I. The T_2 times calculated according to Supplementary Eq. 9 are given in the last row of Supplementary Table 1 and are also shown by the dashed lines in Fig. 4 of the main text. These estimates are based on the maximum possible effect of the heteronuclear coupling. However, comparing them to the $T_{2,\mathrm{zz+ff}}$ values we see that the heteronuclear interaction reduces T_2 times by no more than 40%. Thus the key role in nuclear spin echo decay (and corresponding reduction of T_2) is played by the homonuclear flip-flops[24].

In these calculations of $T_{2,\mathrm{zz+ff+IS}}$ we take into account only the flip-flops of the $S_z = \pm 1/2$ states and the same chemical composition (same values of ρ) as for the studied dots, which corresponds to a hypothetical quantum dot where first-order quadrupolar shifts are strongly inhomogeneous, while second-order shifts are homogeneous or absent. In real self-assembled dots the first-order shifts are strongly inhomogeneous, but the flip-flops of the $\pm 1/2$ states may be suppressed only partially and to a different degree for different isotopes. Thus we may have an intermediate case between cases (i) and (ii), making exact calculation of the nuclear T_2 difficult. But as demonstrated above, one can obtain both the upper and lower bounds on T_2: $T_{2,\mathrm{zz+ff+IS}} < T_2 < T_{2,\mathrm{zz}}$.

The observation of $T_2 \approx T_{2,\mathrm{zz}}$ for ^{75}As nuclei in strained dots is then interpreted as strong suppression of the homonuclear flip-flops due to strongly inhomogeneous second-order quadrupolar shifts of arsenic (resulting from atomic-scale alloy disorder). By contrast for Ga and In isotopes $T_2 < T_{2,\mathrm{zz}}$ is observed in strained dots, implying weaker suppression of the homonuclear flip-flop due to smaller second-order shifts.

Previously the T_2 values calculated using the technique of Ref.[11] were shown to deviate by less than 25% from the experimental values[11]. As expected the values of $T_{2,\mathrm{zz+ff+IS}}$ calculated in the present work are in good agreement with earlier spin-echo decay measurements on ^{75}As [Refs.[18–20]] and ^{71}Ga [Refs.[25,26]] in lattice matched quantum wells and dots (see triangles in Fig. 4 of the main text) where second-order quadrupolar effects are negligible. Somewhat faster than predicted decay of the ^{71}Ga echo [Refs.[25,26]] was possibly due to the non-selective nature of the measurements resulting in additional echo decay due to residual first-order quadrupolar shifts.

---

[1] Puebla, J., Chekhovich, E. A., Hopkinson, M., Senellart, P., Lemaitre, A., Skolnick, M. S., and Tartakovskii, A. I., Dynamic nuclear polarization in InGaAs/GaAs and GaAs/AlGaAs quantum dots under nonresonant ultralow-power optical excitation. *Phys. Rev. B* **88**, 045306 (2013).

[2] Chekhovich, E. A., Kavokin, K. V., Puebla, J., Krysa, A. B., Hopkinson, M., Andreev, A. D., Sanchez, A. M., Beanland, R., Skolnick, M. S., and Tartakovskii, A. I., Structural analysis of strained quantum

dots using nuclear magnetic resonance. *Nature Nanotech.* **7**, 646–650 (2012).

[3] Chekhovich, E. A., Glazov, M. M., Krysa, A. B., Hopkinson, M., Senellart, P., Lemaitre, A., Skolnick, M. S., and Tartakovskii, A. I., Element-sensitive measurement of the hole-nuclear spin interaction in quantum dots. *Nat. Phys.* **9**, 74–78 (2013).

[4] Gammon, D., Efros, A. L., Kennedy, T. A., Rosen, M., Katzer, D. S., Park, D., Brown, S. W., Korenev, V. L., and Merkulov, I. A., Electron and nuclear spin interactions in the optical spectra of single GaAs quantum dots. *Phys. Rev. Lett.* **86**, 5176–5179 (2001).

[5] Tartakovskii, A. I., Wright, T., Russell, A., Fal'ko, V. I., Van'kov, A. B., Skiba-Szymanska, J., Drouzas, I., Kolodka, R. S., Skolnick, M. S., Fry, P. W., Tahraoui, A., Liu, H.-Y., and Hopkinson, M., Nuclear spin switch in semiconductor quantum dots. *Phys. Rev. Lett.* **98**, 026806 (2007).

[6] Lai, C. W., Maletinsky, P., Badolato, A., and Imamoglu, A., Knight-field-enabled nuclear spin polarization in single quantum dots. *Phys. Rev. Lett.* **96**, 167403 (2006).

[7] Eble, B., Krebs, O., Lemaitre, A., Kowalik, K., Kudelski, A., Voisin, P., Urbaszek, B., Marie, X., and Amand, T., Dynamic nuclear polarization of a single charge-tunable InAs/GaAs quantum dot. *Phys. Rev. B* **74**, 081306 (2006).

[8] Braun, P.-F., Urbaszek, B., Amand, T., Marie, X., Krebs, O., Eble, B., Lemaitre, A., and Voisin, P., Bistability of the nuclear polarization created through optical pumping in $In_{1-x}Ga_xAs$ quantum dots. *Phys. Rev. B* **74**, 245306 (2006).

[9] Chekhovich, E. A., Krysa, A. B., Skolnick, M. S., and Tartakovskii, A. I., Light-polarization-independent nuclear spin alignment in a quantum dot. *Phys. Rev. B* **83**, 125318 (2011).

[10] Haase, J. and Conradi, M. S., Sensitivity enhancement for NMR of the central transition of quadrupolar nuclei. *Chemical Physics Letters* **209**, 287 – 291 (1993).

[11] Haase, J. and Oldfield, E., Spin-echo behavior of nonintegral-spin quadrupolar nuclei in inorganic solids. *Journal of Magnetic Resonance, Series A* **101**, 30 – 40 (1993).

[12] Van Vleck, J. H., The dipolar broadening of magnetic resonance lines in crystals. *Phys. Rev.* **74**, 1168–1183 (1948).

[13] Abragam, A., *The principles of Nuclear Magnetism.* Oxford University Press, London (1961).

[14] de Sousa, R. and Das Sarma, S., Theory of nuclear-induced spectral diffusion: Spin decoherence of phosphorus donors in Si and GaAs quantum dots. *Phys. Rev. B* **68**, 115322 (2003).

[15] Witzel, W. M. and Das Sarma, S., Quantum theory for electron spin decoherence induced by nuclear spin dynamics in semiconductor quantum computer architectures: Spectral diffusion of localized electron spins in the nuclear solid-state environment. *Phys. Rev. B* **74**, 035322 (2006).

[16] Yao, W., Liu, R.-B., and Sham, L. J., Theory of electron spin decoherence by interacting nuclear spins in a quantum dot. *Phys. Rev. B* **74**, 195301 (2006).

[17] Slichter, C. P., *Principles of Magnetic Resonance, Third Edition.* Springer-Verlag, Berlin (1989).

[18] Kondo, Y., Ono, M., Matsuzaka, S., Morita, K., Sanada, H., Ohno, Y., and Ohno, H., Multipulse operation and optical detection of nuclear spin coherence in a GaAs/AlGaAs quantum well. *Phys. Rev.*

Lett. **101**, 207601 (2008).

[19] Ono, M., Sato, G., Ishihara, J., Matsuzaka, S., Ohno, Y., and Ohno, H., Nuclear spin coherence time in a strained GaAs quantum well. *AIP Conference Proceedings* **1399**, 685–686 (2011).

[20] Ishihara, J., Ono, M., Sato, G., Matsuzaka, S., Ohno, Y., and Ohno, H., Magnetic field dependence of quadrupolar splitting and nuclear spin coherence time in a strained (110) GaAs quantum well. *Japanese Journal of Applied Physics* **50**, 04DM03 (2011).

[21] Davies, J. H., Elastic and piezoelectric fields around a buried quantum dot: A simple picture. *Journal of Applied Physics* **84**, 1358–1365 (1998).

[22] Kambe, K. and Ollom, J. F., Dipolar broadening of the central line of a magnetic resonance for half-integral spin. *Journal of the Physical Society of Japan* **11**, 50–52 (1956).

[23] Recchia, C. H., Gorny, K., and Pennington, C. H., Gaussian-approximation formalism for evaluating decay of NMR spin echoes. *Phys. Rev. B* **54**, 4207–4217 (1996).

[24] Ota, T., Yusa, G., Kumada, N., Miyashita, S., Fujisawa, T. and Hirayama, Y., Decoherence of nuclear spins due to dipole-dipole interactions probed by resistively detected nuclear magnetic resonance. *Applied Physics Letters* **91**, 193101 (2007).

[25] Sanada, H., Kondo, Y., Matsuzaka, S., Morita, K., Hu, C. Y., Ohno, Y., and Ohno, H., Optical pump-probe measurements of local nuclear spin coherence in semiconductor quantum wells. *Phys. Rev. Lett.* **96**, 067602 (2006).

[26] Makhonin, M. N., Kavokin, K. V., Senellart, P., Lemaitre, A., Ramsay, A. J., Skolnick, M. S., and Tartakovskii, A. I., Fast control of nuclear spin polarization in an optically pumped single quantum dot. *Nature Materials* **10**, 848 (2011).
